# Translation and cultural adaption of the control preference scale across various care settings in a Danish hospital

**DOI:** 10.1186/s41687-024-00771-3

**Published:** 2024-08-12

**Authors:** Bettina Mølri Knudsen, Karina Dahl Steffensen

**Affiliations:** 1 Center for Shared Decision Making, Lillebaelt Hospital - University Hospital of Southern Denmark, Beriderbakken 4, Vejle, Denmark; 2 Department of Regional Health Research, Faculty of Health Sciences, University of Southern Denmark, Odense, Denmark

**Keywords:** Shared decision making, Preference, Preferred role, Control preference scale, Translation, Content validity

## Abstract

**Background:**

In recent decades, there has been a growing emphasis on involving patients in healthcare decision-making, driven by political, ethical, and research considerations. Although patient involvement is associated with improved health outcomes, understanding patient preferences regarding their role in decision-making is crucial for effective interventions. The Control Preferences Scale (CPS) measures patient preferences along a continuum from passive to active participation. However, its application in Denmark necessitates translation and cultural adaptation.

**Methodology:**

This study aimed to translate and culturally adapt the CPS for Danish use across diverse healthcare settings: acute care, cancer care, elective surgery, chronic medical treatment, and parental involvement in pediatric care. Following a cross-sectional design, the translation process was systematically planned and executed using Beaton’s guidelines, including the five stages: forward and back translation, synthesis, expert review, and pre-testing.

**Results:**

The translation and adaption process was carried out successfully. Few linguistic challenges were identified and resolved by the expert review. The findings of the pre-testing indicated high acceptability and usability of the adapted CPS among 152 Danish patients and parents. The collaborative role emerged as the most preferred across settings (69.8%), with passive roles more prevalent among cancer patients (30%) and parents waiting with their child to see a pediatrician (23.3%). Notable, more women preferred collaborative or active roles (83.9%) than men (73.9%). The content validity assessment yielded positive feedback, affirming the relevance and comprehensiveness of the CPS.

**Conclusions:**

In summary, the adaptation and validation of the CPS for Danish use proved successful, providing a valuable tool for assessing patient’s role preferences in healthcare decision-making. However, future studies are recommended to ensure construct validity and reliability through psychometric testing.

**Supplementary Information:**

The online version contains supplementary material available at 10.1186/s41687-024-00771-3.

## Background

Over the past two decades, there has been a growing interest in initiatives geared towards involving citizens and patients in healthcare to foster active participation in decisions about their treatment and care. This perspective is shaped by political, ethical, and research considerations [1, 2]. While patient involvement generally correlates with improved health outcomes and higher satisfaction levels [3], the promotion of shared decision making (SDM) faces challenges arising from divergent attitudes, preferences and expectations among physicians and patients [4–6].

Several studies have explored patients’ preferences regarding their role in decision-making [3, 7, 8], recognizing that these preferences can impact the efficacy of interventions designed to enhance patient involvement. Evaluating the alignment between preferred and actual roles stands as a crucial outcome measure for such interventions [9]. Thus, formal assessment of these preferences is imperative. Furthermore, comparing role preferences across diverse patient groups may reveal cultural disparities, offering valuable insights for initiatives aimed at enhancing patient-physician communication [10, 11].

Patient involvement in healthcare decision-making is often conceptualized along a continuum from passive to active participation [12], with the Control Preferences Scale (CPS) frequently employed to assess patient preferences regarding their involvement in decision-making along this continuum [13]. However, since the CPS was originally developed in English, its application in a Danish context necessitates translation and cultural adaptation. Therefore, the objective of this study was to translate and culturally adapt the CPS for use in Denmark. To enhance the scale’s future applicability, the translation underwent testing across five different settings, including acute care, cancer care, elective surgery, chronic medical treatment, and parental involvement in pediatric care.

## Methods

### Design

A cross-sectional design was employed for the translation and cultural adaption process of the CPS from English to Danish. This process followed the systematic approach outlined by Beaton’s et al. guidelines [14], comprising five stages: forward-translation, synthesis, back-translation, expert committee review and pre-testing. Additionally, the assessment of the translation process was conducted using the COSMIN study design checklist for patient-reported outcome measurement instruments [15].

### The control preference scale

**Fig. 1:**
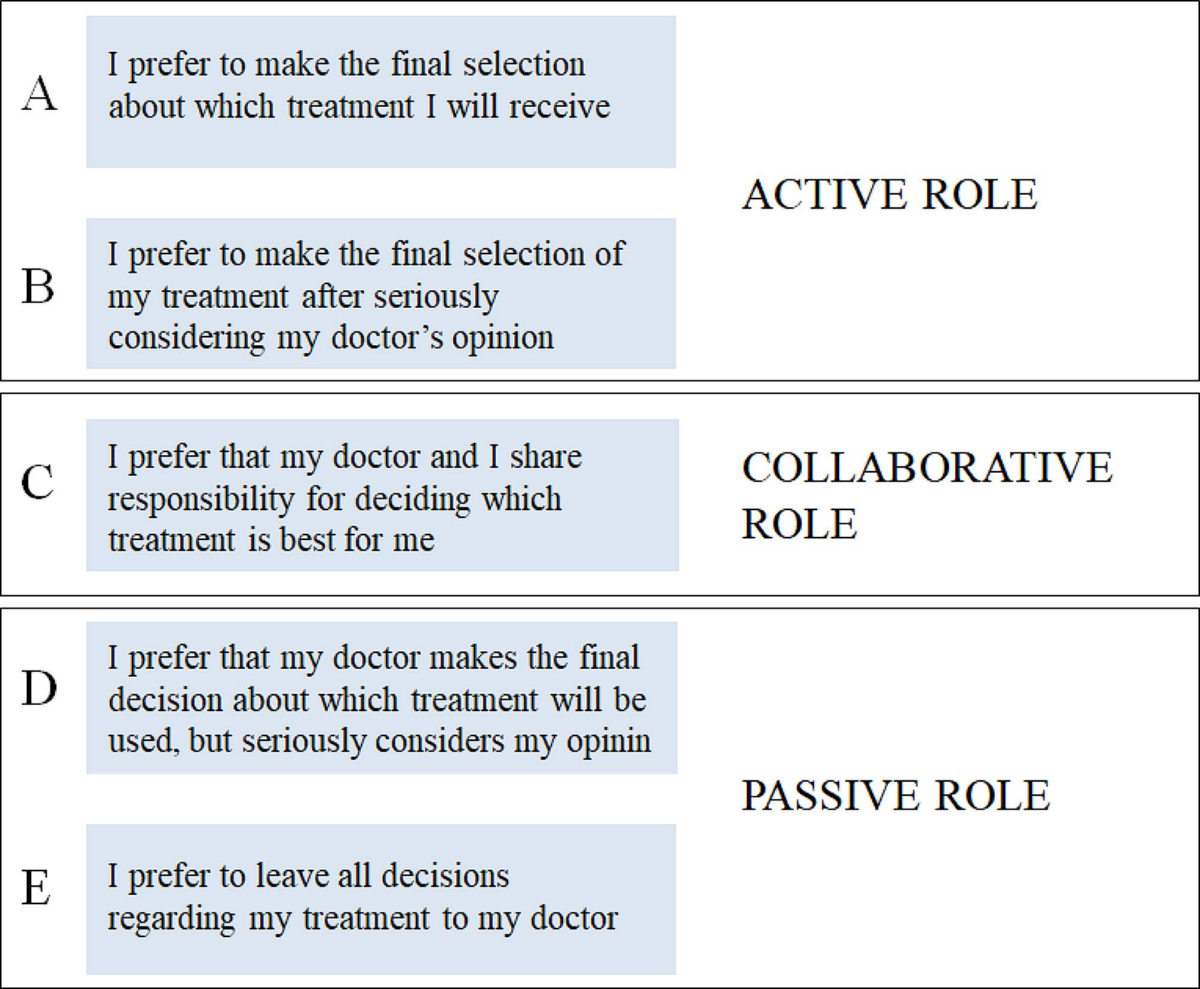
Five decisional roles grouped into three

The CPS, developed by Degner et al. serves to assess the level of control patients desire in decision-making regarding their treatment and is shown to be clinically relevant, easily administered, and generates valid data on patients’ preferences in medical decision-making [13]. The CPS consists of five cards depicting five distinct decisional roles patients may assume during decision-making moments. Each role is illustrated by a descriptive statement (from A to E), optionally accompanied by cartoons, and can be grouped into three overarching roles: active, collaborative and passive [12] (Fig. 1).

The scale offers flexibility in administration methods. Degner et al. recommend two main approaches for clinical settings: the fixed-order or pick-one approach [13]. In this study, the pick-one approach was adopted, excluding the use of the cartoons.

### Translation process—stages I–IV

Using Beaton’s guidelines, the research team followed the five distinct stages, all coordinated by the project lead. At stage I, the original CPS underwent forward translation by two bilingual translators, both native Danish speakers: T1 a physician and T2 a linguist. Working independently, they produced individual translations. At stage II, T1 and T2 collaborated to reconcile a final version, synthesizing their translations and referring to the original questionnaire.

At stage III, the synthesized Danish version underwent back translation by two native English-speaking translators, B1 and B2, blinded to the original English version and working independently. This step ensured that the meaning of the Danish version was accurately reflected in the back-translated version. Both translators had academic backgrounds currently working in health care. After the back-translations, at stage IV, an expert committee reviewed all versions and discussed any discrepancies. The expert committee comprised all translators, the project lead and two experts in quantitative methods and SDM. At this stage, equivalence in semantics and intended content of each role were evaluated.

### Pre-testing—stage V

In the final stage, the pretest was carried out. According to Beaton et al. 30–40 respondents from the target group are recommended [14]. As the CPS can be used in different settings, it was decided to include approximately 30 respondents from each setting.

### Informants and recruitment for pre-testing

To ensure representation of diverse perspectives, patients of varying age, gender and health conditions were personally approached during their visits to a Danish hospital in the Region of Southern Denmark. Patients were recruited across five different settings, (1) at the emergency department awaiting acute care, (2) at the oncology department awaiting consultation with an oncologist, (3) at the orthopedic department before or after elective hip or knee surgery, (4) at the medical department awaiting consultation with a physician regarding chronic disease management, and (5) in the pediatric department were parents were waiting with their child for treatment.

Approached participants were in the waiting rooms before their scheduled consultation and were not informed about the study before approach. First, each participant received an explanation of the study. Second, on one page, participants were asked to indicate their gender and age (and the age of the child if the participant was a parent) and complete the CPS. On completion, they were asked to articulate their thoughts aloud while evaluating the five roles of the CPS, choosing the one that most closely matched their preferred role for the upcoming consultation with the physician. They were encouraged to articulate whether the statements reflected their preference or if their ideal role might fall between two statements. The interviews were conducted by a researcher and a nurse, dressed in civilian clothes and introducing themselves as “interviewers”. The interviewers took notes during the interview. Feedback from this stage was reviewed and analysed for content validity assessment.

### Statistical analysis

The CPS was treated as an ordinal variable with five categories describing preferences in decision making, ranging from completely active to completely passive. In addition, we categorized the five statements into three groups of decision preferences reflecting “active”, “passive” and “collaborative” roles based on participants’ choices (see Fig. 1). Descriptive statistics were used to analyze the frequencies for the three categories of CPS and the age distribution within the predefined groups, with the responses entered into Excel and analyzed descriptively.

## Results

### Translation process stages I-IV

The translation process was planned and executed between March and May 2022. Based on the COSMIN guidelines, an assessment of the translation process revealed high quality (see Supplementary Table 1).

Stages I-III went smoothly without encountering any issues. However, during Stage IV, discrepancies in the wording occurred when comparing the two back-translations to the original scale in the expert committee. The original scale used the term “selection” in the first two statements (A and B) and the term “decision” in the last two statements (D and E). It was decided to use the Danish word “beslutning” (in English: decision) consistently for all four statements in the translated version, which corresponds to the prevailing terminology in Denmark.

In addition, adjustments were made to the translation of statements B–E. “My doctor” was translated as “the doctor” (Danish: lægen) to capture a broader context, which is particularly relevant in a hospital setting, as the use of “my doctor” evokes strong associations with the patient’s general practitioner. Also, the term “responsibility” was discussed in statement C. The committee debated whether it might be perceived as inappropriate and too harsh to place responsibility on the patients. Given its infrequent use in Danish medical contexts and concerns about legal and ethical implications of transferring responsibility to the patient, a pilot test was conducted with six patients and four physicians. Two translation options of the Danish CPS were presented to them, which differed in the wording of statement C. In the first option, the term “responsibility” was retained (in Danish: ansvar); the second option emphasized being part of a shared decision making process about what treatment is best for the patient (in Danish: at være sammen om at beslutte). The participants were interviewed. One physician favored the term “responsibility” but only if used consistently in all statements for reasons of coherence. The rest of the participants, both patients and physicians, were in favor of the second option and described the term “responsibility” as discouraging, distancing and too formal. Consequently, the expert committee decided in favor of option 2. This decision, influenced by participant feedback, aligns well with the cultural practice of shared decision making in Denmark, where patients and physicians collaborate in treatment decisions without using the term “responsibility”. The final Danish version can be found in Table 1.

**Table 1:** The five statements in the Danish version of CPS

A Jeg foretrækker selv at træffe den endelige beslutning om, hvilken behandling jeg skal have
B Jeg foretrækker selv at træffe den endelige beslutning om min behandling efter nøje at have overvejet lægens holdning
C Jeg foretrækker, at lægen og jeg er sammen om at beslutte, hvilken behandling der er bedst for mig
D Jeg foretrækker, at lægen, efter nøje at have overvejet min holdning, træffer den endelige beslutning om hvilken behandling, der skal anvendes
E Jeg foretrækker at overlade alle beslutninger vedrørende min behandling til lægen

### Stage V—pre-testing

Participants for the pre-testing were recruited from August to September 2022. A total of 122 patients and 30 parents were asked to participate in the study. Table 2 shows the characteristics of the participants. All 152 participants completed the CPS and provided feedback to the interviewer when completing the CPS.

**Table 2:** Characteristics of participants

Variable	Study participants (*n* = 152)	
	*n*	%
**Adult patients and parents (** ***n*** ** = 152)**		
Age (years) (mean ± SD)	53.97 ± 18.80	
18–44	52	34.2
45–64	46	30.3
65–84	50	32.9
> 85	4	2.6
**Gender**,** adult patients and parents**		
Male	65	42.8
Female	87	57.2
**Children (** ***n*** ** = 30)**		
Age (years) (mean ± SD)	9.32 ± 4.32	

### Control preference distribution

The most commonly preferred role was the collaborative role, regardless of setting. Among those expressing a preference for either an active or passive role, the passive role was more prevalent than the active role, especially among cancer patients waiting to see their oncologist (9 patients, 30%), emergency department patients waiting for acute care (7 patients, 21.9%), and parents waiting with their child to see a pediatrician (7 parents, 23.3%). Table 3 shows the distribution of role preferences within the three CPS groups categorized by age, gender and setting.

A higher percentage of women than men indicated a preference for either a collaborative (62 women, 71.3%) or an active role (11 women, 12.6%), indicating a statistically significant association between gender and role preference (p-value of 0.0028). Preference for a collaborative role increased with age, peaking in the 65–84 year age group (76.0%), and then declined among patients aged 85+, where the majority (3 patients, 75.0%) indicated a preference for a passive role.

**Table 3:** Tabulation of the three CPS groups “active”, “passive”, and “collaborative” according to age, gender and settings

	Active	Collaborative	Passive
	*N*/%	*N*/%	*N*/%
**Age group**,** adult patients and parents (*n* = 152)**			
18–44 (*n* = 52)	9/17.3	33/63.5	10/19.2
45–64 (*n* = 46)	3/6.5	34/73.9	9/19.6
65–84 (*n* = 50)	3/6.0	38/76.0	9/18.0
> 85 (*n* = 4)	–	1/25.0	3/75.0
**Gender (** ***n*** ** = 152)**			
Male (*n* = 65)	4/6.2	44/67.7	17/26.1
Female (*n* = 87)	11/12.6	62/71.3	14/16.1
**Setting**			
Emergency department, acute care	4/12.5	21/65.6	7/21.9
Oncology department, cancer care	2/6.7	19/63.3	9/30.0
Orthopedic department, elective surgery	3/10.0	23/76.7	4/13.3
Medical department, chronic care management	4/13.3	22/73.4	4/13.3
Pediatric department, treatment/care of child	2/6.7	21/70.0	7/23.3

### Content validity assessment

Overall, the participants found the CPS to be straightforward and user-friendly. The language was described as simple, accessible, and easy to understand. Even a patient from Afghanistan, awaiting consultation for a chronic disease, found the questionnaire understandable, although it required some additional reading time. While a few participants needed to reread the items to fully grasp their nuances before responding, the majority found it manageable.

A few participants suggested minor adjustments to improve clarity in the nuances of the five items, aiming to prevent potential misunderstandings. However, these suggestions were limited in number and consistency. Therefore, they did not warrant actual modifications of the Danish CPS.

Regarding content validity, participants provided overwhelmingly positive feedback. Many expressed that the range of alternative roles presented in the CPS prompted them to consider different perspectives on participating in decision-making. They appreciated the opportunity to express their preferences before engaging in the medical encounter, highlighting the value of the questionnaire in facilitating patient engagement in healthcare decisions.

## Discussion

In this study, the CPS was successfully translated and adapted into Danish, and the content validity assessment showed high acceptability and usability of the adapted CPS among Danish patients and parents across five different healthcare settings.

The CPS has been used in an non-validated Danish version in a previous survey among healthy Danes where participants were asked to express their hypothetical preferences in various settings [16]. The survey revealed that a significant majority of Danes favored a collaborative role in patient-physician encounters. However, in acute settings, 67% of the Danes preferred a passive role [16], which is in contrast to the 21.9% of patients in our acute setting preferring a passive role. The survey among healthy Danes was conducted several years ago, which may explain this shift away from the passive role. Additionally, Lechner et al. [8] found a significantly higher frequency of preference for a passive role among the older age groups, and a higher frequency of preference for an active role among younger patients in 2016. Our study results diverge from this pattern, as all age groups up to the age of 84 preferred a collaborative role. This suggest a shift towards a more collaborative role among all age groups over the last 8–10 years, possibly influenced by a growing emphasis on patient deliberation and patient-centered care. Nevertheless, patients appears to have a stronger desire to communicate about their treatment and care.

The CPS captures the patients’ preferred preferences before seeing the physician. However, it is essential to determine whether patients are actually involved in decision making. Within the Danish healthcare context, the annual Patient Experience Survey (LUP) [17] is an evaluation conducted nationwide in Denmark. This survey serves as a cornerstone for assessing patient satisfaction and identifying areas for improvement within healthcare. Of particular significance within the LUP is patients’ reported experiences of involvement in decisions about their treatment. Strikingly, this item concerning shared decision making consistently records the lowest scores across various patient groups, including planned admissions, emergency admissions, and planned outpatient visits. It highlights a gap between the observed low scores and the principles of patient-centered care and raises the question of whether the current measurement approach adequately captures the multifaceted nature of patient involvement or if it indicates that there is room for improvement in our efforts to engage patients effectively. Simply assessing whether patients perceive themselves as involved in decision-making may not provide a comprehensive understanding of their true experiences and preferences. One potential approach for addressing this gab is the integration of the CPS into the LUP to gain deeper insights into patients’ expectations and experiences regarding decision-making processes. This will enable us to assess whether patients’ preference for the level of involvement aligns with their actual experiences. Moreover, the content validity assessment conducted in this study showed that the participants highly valued the opportunity to express their preferences, thereby fostering a heightened awareness of their ability to play an active role in their treatment and care.

A review of the literature shows that the CPS has been translated into Arabic [18], Greek [19], Japanese [20], and Swedish [21]. All studies involved breast cancer patients, with data collected between 2009 and 2015. Interestingly, all four studies indicated that breast cancer patients exhibited a higher preference for a passive role compared to what was observed among cancer patients in our study. Nevertheless, it is crucial to note that our study’s data are over ten years more recent, and cultural factors may also influence patients’ preferences. Despite this difference, the translations performed in these studies were found to be highly consistent, demonstrating the suitability of CPS for assessing patients’ preferred role across different cultural contexts.

The sample size in our study aligns with the recommendations of Degner et al. for the pre-test [13]. However, there is limited representation of participants in the youngest and oldest age groups, and quantitative studies with larger samples are needed to validate our findings in these age groups. Additionally, as participants were approached randomly, our sample likely comprise patients with varying disease durations and stages, from recent diagnosis to many years of illness. While our study’s results are consistent across all age groups, these factors may influence patients’ preferred role. To ensure construct validity and reliability, future studies should conduct psychometric testing of responses to the Danish CPS. This will provide a comprehensive evaluation of the scale’s performance and strengthen the robustness of our findings.

## Conclusions

In conclusion, adaptation and validation of the CPS for a Danish context were successful, establishing its content validity and readiness for use. Patient feedback indicated that the CPS was relevant, comprehensive, and valuable for expressing their preferences of keeping, sharing or giving away control over decision making to the physician before engaging in the medical encounter. This underscores the value of the questionnaire in facilitating patient engagement in healthcare decisions. However, to further strengthen its reliability and validity, a future study testing the psychometric properties of the Danish CPS is recommended, focusing on construct validity and reliability assessments.

### Electronic supplementary material

Below is the link to the electronic supplementary material.

Supplementary Material 1

## Data Availability

The datasets used and/or analysed during the current study are available from the corresponding author on reasonable request.

## Abbreviations

SDM: Shared decision making
CPS: Control Preferences Scale
LUP: Patient Experience Survey

## References

[1] Steffensen KD et al (2022) Implementation of patient-centred care in Denmark: the way forward with shared decision-making. Zeitschrift für Evidenz Fortbildung Und Qualität Im Gesundheitswesen 171:36–41 35606311 10.1016/j.zefq.2022.04.005

[2] Coulter A (2018) National strategies for implementing shared decision making. Bertelsmann Stiftung

[3] Stacey D et al (2024) Decision aids for people facing health treatment or screening decisions. Cochrane Database Syst Rev (1) 10.1002/14651858.CD001431.pub6 PMC10823577 38284415

[4] Légaré F et al (2014) Interventions for improving the adoption of shared decision making by healthcare professionals. Cochrane Database Syst Rev (9). 10.1002/14651858.CD006732.pub3 25222632

[5] Scholl I et al (2018) Organizational-and system-level characteristics that influence implementation of shared decision-making and strategies to address them—a scoping review. Implement Sci 13(1):1–22 29523167 10.1186/s13012-018-0731-z PMC5845212

[6] Joseph-Williams N, Elwyn G, Edwards A (2014) Knowledge is not power for patients: a systematic review and thematic synthesis of patient-reported barriers and facilitators to shared decision making. Patient Educ Couns 94(3):291–309 24305642 10.1016/j.pec.2013.10.031

[7] Arora NK, McHorney CA (2000) Patient preferences for medical decision making: who really wants to participate? Medical care 335–341 10.1097/00005650-200003000-00010 10718358

[8] Lechner S et al (2016) Control preferences in treatment decisions among older adults—results of a large population-based study. J Psychosom Res 86:28–33 27302543 10.1016/j.jpsychores.2016.05.004

[9] Kasper J et al (2008) Informed shared decision making about immunotherapy for patients with multiple sclerosis (ISDIMS): a randomized controlled trial. Eur J Neurol 15(12):1345–1352 19049552 10.1111/j.1468-1331.2008.02313.x

[10] Chewning B et al (2012) Patient preferences for shared decisions: a systematic review. Patient Educ Couns 86(1):9–18 21474265 10.1016/j.pec.2011.02.004 PMC4530615

[11] Singh JA et al (2010) Preferred roles in treatment decision making among patients with cancer: a pooled analysis of studies using the Control Preferences Scale. Am J Manag Care 16(9):688 20873956 PMC3020073

[12] Degner LF, Sloan JA (1992) Decision making during serious illness: what role do patients really want to play? J Clin Epidemiol 45(9):941–950 1432023 10.1016/0895-4356(92)90110-9

[13] Degner LF, Sloan JA, Venkatesh P (1997) The control preferences scale. Can J Nurs Res Arch 21–44 9505581

[14] Beaton DE et al (2000) Guidelines for the process of cross-cultural adaptation of self-report measures. Spine 25(24):3186–3191 11124735 10.1097/00007632-200012150-00014

[15] Mokkink LB et al (2010) The COSMIN checklist for assessing the methodological quality of studies on measurement properties of health status measurement instruments: an international Delphi study. Qual Life Res 19:539–549 20169472 10.1007/s11136-010-9606-8 PMC2852520

[16] MandagMorgen, TrygFonden (2016) Sundhedsvæsenet ifølge danskerne. Report, Mandag Morgen and Trygfonden. https://www.tryghed.dk/viden/publikationer/sundhed/sundhedsvaesenet-ifoelge-danskerne. Accessed 4 June 2024

[17] Center for Patientinddragelse. Landsdækkende Undersøgelse af Patientoplevelser. https://www.regionh.dk/patientinddragelse/LUP/Sider/default.aspx. Accessed 4 June 2024

[18] Obeidat R (2015) Decision-making preferences of Jordanian women diagnosed with breast cancer. Support Care Cancer 23:2281–2285 25576431 10.1007/s00520-014-2594-4

[19] Almyroudi A et al (2011) Decision-making preferences and information needs among Greek breast cancer patients. Psycho‐Oncology 20(8):871–879 20623805 10.1002/pon.1798

[20] Azuma K et al (2021) Development of Japanese versions of the control preferences scale and information needs questionnaire: role of decision-making and information needs for Japanese breast cancer patients. Patient Prefer Adherence 1017–1026 10.2147/PPA.S295383 PMC8140924 34040355

[21] Wallberg B et al (2000) Information needs and preferences for participation in treatment decisions among Swedish breast cancer patients. Acta Oncol 39:467–476 11041108 10.1080/028418600750013375

