# Supplementary material for: Translation and cultural adaption of the control preference scale across various care settings in a Danish hospital
Source: J Patient Rep Outcomes. 2024 Aug 12;8:91. doi: 10.1186/s41687-024-00771-3 (PMC11319534; doi:10.1186/s41687-024-00771-3)
Supplement: Supplementary file 1 — Supplementary Material 1 [file 41687_2024_771_MOESM1_ESM.docx]

**Supplementary table 1. Translation process**

| **Design requirements** | **Very good** | **Adequate** | **Doubtful** | **Inadequate** |
| --- | --- | --- | --- | --- |
| 1. Describe both the original language in which the PROM was developed, the source language (if different from the original language) and the language in which the PROM will be translated | **Original language, source language and target language will be described** |  |  | Source language will NOT be described |
| 2. Ensure that the items will be translated forward and backward | **Multiple forward and multiple backward translations** | Multiple forward translations but one backward translation | One forward and one backward translation | Only a forward translation |
| 3. Ensure that both forward translators have a mother tongue in the target language in which the PROM will be translated | **Both forward translators have a mother tongue in the target language in which the PROM will be translated** |  | Only one of the forward translators a mother tongue in the target language in which the PROM will be translated | Both forward translators don´t have a mother tongue in the target language |
| 4. Ensure that one of the forward translators has expertise in the diseases involved, and in the construct measured by the PROM; the other forward translators is naïve on the construct measured by the PROM | **One of the forward translators has expertise on disease and construct to be measured, other translator is naïve** | Unclear what expertise of both forward translators with respect to disease or construct | Both forward translators are either both experts with respect to disease or construct, or both naïve with respect to disease or construct |  |
| 5. Ensure that both backward translators have a mother tongue in the original or source language | **Both backward translators have a mother tongue in the source language in which the PROM will be translated** |  | Only one of the backward translators a mother tongue in the source language in which the PROM will be translated | Both backward translators don’t have a mother tongue in the source language |
| 6. Ensure that both backward translators are naïve in the disease involved and the construct to be measured | **Both backward translators will be naïve in the disease involved and the construct to be measured** | Unclear if both backward translators will be naïve in the disease involved and the construct to be measured |  |  |
| 7. Ensure that the translators will work independently from each other | **Translators will work independent** | Assumable that the translators will work independent | Unclear whether translators will work independent | Translators will NOT work independent |
| 8. Provide a clear description on how differences between the original and translated versions will be resolved | **Adequate description of how differences between translators will be resolved** | Poorly or NOT described how differences between translators will be resolved |  |  |
| 9. Ensure that the translation will be reviewed by a committee (including the original developers of the PROM) | **Translation will be reviewed by a committee (involving other people than the translators, e.g. the original developers)** | Translation will NOT be reviewed by (such) a committee |  |  |
| 10. Write a feedback report of the translation process | **Feedback report will be written** |  | No feedback report will be written |  |
| 11 Perform a pilot study (e.g. cognitive interview study) to check (1) the relevance of each item for the patients’ experience with the condition, AND (2) the comprehensiveness of the PROM, AND (3) the comprehensibility of the PROM instructions, items, response options, and recall period | **Widely recognized or well justified method for qualitative research will be used to assess the three aspects** | Only quantitative (survey) method(s) will be used or assumable that the method used will be appropriate but not clearly described, but all three aspects will be assessed | Not clear if patients will be asked whether each item is relevant AND comprehensible AND whether items together are comprehensive, or doubtful whether the method will be appropriate | Method used are not appropriate or patients will not be asked about the relevance, comprehensiveness or comprehensibility of all items (Ro12.B box 1) |
| 12. Perform the pilot study in a patient population representing the target population | **The study will be performed in a sample representing the target population** | Assumable that the study will be performed in a sample representing the target population | Doubtful whether the study will be performed in a sample representing the target population | Study will NOT be performed in a sample representing the target population |

From COSMIN study design checklist for patient-reported outcome measurement instruments (Mokkink et al. 2019)
